# Targeting Notch enhances the efficacy of ERK inhibitors in BRAF-V600E melanoma

**DOI:** 10.18632/oncotarget.12078

**Published:** 2016-09-16

**Authors:** Clemens Krepler, Min Xiao, Minu Samanta, Adina Vultur, Hsin-Yi Chen, Patricia Brafford, Patricia I. Reyes-Uribe, Molly Halloran, Thomas Chen, Xu He, Denitsa Hristova, Qin Liu, Ahmed A. Samatar, Michael A. Davies, Katherine L. Nathanson, Mizuho Fukunaga-Kalabis, Meenhard Herlyn, Jessie Villanueva

**Affiliations:** ^1^ The Wistar Institute, Melanoma Research Center, Philadelphia, PA, USA; ^2^ Discovery Oncology Merck Research Laboratories, Boston, MA, USA; ^3^ Melanoma Medical Oncology and Systems Biology University of Texas MD Anderson Cancer Center, Houston, TX, USA; ^4^ Division of Medical Genetics and The Abramson Cancer Center, University of Pennsylvania School of Medicine, Philadelphia PA, USA

**Keywords:** melanoma, targeted therapy resistance, ERK, BRAF, Notch

## Abstract

The discovery of activating BRAF mutations in approximately 50% of melanomas has led to the development of MAPK pathway inhibitors, which have transformed melanoma therapy. However, not all BRAF-V600E melanomas respond to MAPK inhibition. Therefore, it is important to understand why tumors with the same oncogenic driver have variable responses to MAPK inhibitors. Here, we show that concurrent loss of PTEN and activation of the Notch pathway is associated with poor response to the ERK inhibitor SCH772984, and that co-inhibition of Notch and ERK decreased viability in BRAF-V600E melanomas. Additionally, patients with low PTEN and Notch activation had significantly shorter progression free survival when treated with BRAF inhibitors. Our studies provide a rationale to further develop combination strategies with Notch antagonists to maximize the efficacy of MAPK inhibition in melanoma. Our findings should prompt the evaluation of combinations co-targeting MAPK/ERK and Notch as a strategy to improve current therapies and warrant further evaluation of co-occurrence of aberrant PTEN and Notch activation as predictive markers of response to therapy.

## INTRODUCTION

Melanoma is the most lethal form of skin cancer. More than 76,000 new cases of melanoma are expected to be diagnosed in the United States this year, with an estimated 9,710 deaths [1]. Even though significant progress has been made treating metastatic melanoma, the 5-year survival rate for patients with distant metastatic disease is ~15%. Therefore, effective and durable therapies for melanoma are urgently needed.

Important progress has been made in recent years identifying the genetic alterations and signaling pathways deregulated in melanoma [2]. Almost 50% of melanomas harbor activating mutations in the *BRAF* oncogene, predominantly V600E, leading to constitutive activation of the mitogen activated protein kinase (MAPK) pathway [3]. Other frequent genomic alterations in melanoma include deletion or inactivating mutations of *PTEN* and *CDKN2A*, amplifications or activating mutations of NRAS, *AKT*, *CCND1*, *MITF*, *KIT*, and *TERT*, among others [2]. *PTEN* deletions co-exist with *BRAF* mutations in approximately 30% of melanomas [4], leading to the concurrent activation of the MAPK and PI3K/AKT pathways. These observations have prompted the development of relatively specific and potent inhibitors against a number of molecules within these two pathways [5].

Although the availability of three FDA-approved MAPK pathway (BRAF and MEK) inhibitors has benefited many patients with BRAF-V600E metastatic melanoma, nearly 30% of BRAF-V600E melanomas do not respond to inhibitors of the MAPK pathway [5]. Furthermore, tumors invariably develop drug resistance and patients ultimately relapse. Even though the mechanisms of resistance are diverse, the MAPK pathway is frequently reactivated in melanomas resistant to these drugs, underscoring the addiction of BRAF mutant melanomas to this pathway [6]. Therefore, it is critical to identify more effective strategies to achieve complete and prolonged inhibition of MAPK signaling in melanoma. ERK inhibition constitutes a promising strategy to offset drug resistance, as blocking ERK would prevent MAPK reactivation driven by bypass mechanisms including RAS, RTKs, BRAF splice variants, and MEK1/2 mutations [7–9]. SCH772984 (SCH984) has been shown to be active in models of acquired resistance to BRAF and MEK inhibitors [8]. Its clinical analogue, MK-8353 (formerly SCH900353), has been evaluated for safety, tolerability, and efficacy in a multi-center clinical trial (ClinicalTrials.gov Identifier: NCT01358331) and other ERK inhibitors such as BVD523 and GDC-0994 are in clinical development [10].

While ERK constitutes a promising target for melanoma therapy, it is unlikely that blocking a single molecule or a single pathway will lead to long-term responses, as tumors can rapidly adapt to pharmacological inhibitors by activating compensatory pathways. Combination therapies will be needed to kill the vast majority of tumor cells in therapy-naïve patients or prevent surviving cells from re-growing. Furthermore, based on previous experience with BRAF and MEK inhibitors, it is plausible that not all BRAF-V600E melanomas are uniformly sensitive to ERK inhibition; hence, it is important to identify molecular markers associated with response and resistance. Preclinical studies have suggested that loss of BIM [11] or Rb [12] is associated with an attenuated response to BRAF inhibitors. Other studies have shown that BRAF inhibition induces PI3K/AKT activation and that PI3K/mTOR inhibitors enhances the anti-melanoma activity of BRAF and MEK inhibitors *in vivo* [13, 14]. Notably, PTEN loss was found to be modestly associated with lower response rates to vemurafenib [6] and patients with PTEN loss had shorter progression free survival (PFS) than patients with normal PTEN in studies with dabrafenib [15].

While combining MAPK and PI3K inhibitors is an attractive strategy for melanoma therapy, the efficacy of this combination is frequently limited by toxicity. We posit that identifying molecular determinants of response to ERK inhibition will be useful to select patients who are most likely to benefit from these drugs. This knowledge will be important in guiding the development of effective strategies to maximize the effects of MAPK inhibitors in melanoma. Here, we have used the ERK inhibitor SCH772984 as a tool to investigate the molecular determinants of resistance to ERK inhibition in BRAF-V600E melanomas and assessed the potential therapeutic value of co-targeting ERK and the Notch pathway.

## RESULTS

### BRAF-V600E melanoma cells respond heterogeneous to ERK inhibition

**Figure 1:**
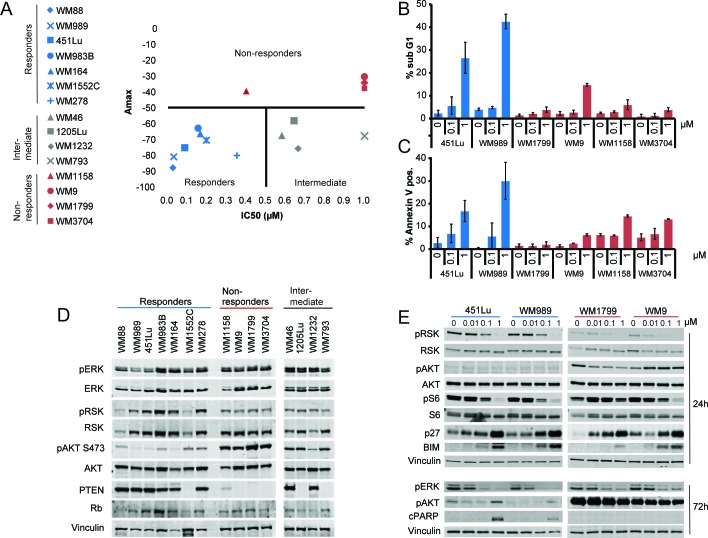
Differential sensitivity of BRAF-V600E melanomas to ERK inhibition **A.** IC50 and maximum inhibitory activity (Amax) in BRAF-V600E melanoma cell lines treated with SCH984 (5×10^−6^ - 10 μM) for 72h. Responders are shown in blue, non-responders in red, and intermediate cell lines in gray. The data represents the mean of three independent experiments; data was normalized to positive (doxorubicin, 10 μM) and negative (DMSO, 0.1 μM) controls. **B.** Two representative responder and all non-responder cell lines were treated with SCH984 for 72h, stained with propidium iodide, and analyzed by flow cytometry. Percent of cells with a sub G1 DNA content indicative of apoptosis are shown; data show mean of three independent experiments +/− SEM. **C.** Annexin V staining to determine apoptosis was performed on cells treated as in B?; mean of three experiments +/− SEM. **D.** Immunoblot analysis of all cell lines grouped based on response to ERK inhibition. Membranes were probed with the indicated antibodies; vinculin was used as loading control. **E.** Two responder and 2 non-responder cell lines were treated with SCH984 at the indicated doses for 24 or 72 h. Total protein lysates were analyzed by immunoblotting; vinculin was used as loading control.

To determine relative sensitivity to ERK inhibition, a panel of BRAF-V600E cells (Supplemental Table S1) was treated for 72 hours with increasing doses of SCH984; IC50 values and maximum inhibitory activity (Amax) were calculated as described in Materials and Methods. While IC50 ranks the cell lines based on potency, Amax ranks the cell lines by efficacy. The combination of these two parameters gives a rigorous measure of the effect that SCH984 has on each cell line (Figure 1A). Based on these parameters, we clustered the melanoma cells into three subgroups: i) Non-responders (Amax < 50%) ii) Responders (Amax > 50% and IC50 < 500 nM) and iii) Intermediate (Amax > 50% but IC50 > 500 nM). Responses to BRAF or MEK inhibitors were similar (Suppl. Figure S1A-B), indicating that this was a general response to MAPK inhibition. Furthermore, whereas SCH984 induced apoptosis in the cells classified as responders, non-responder cells were significantly less affected (Figure 1B-1C).

As PTEN has been previously implicated in resistance to BRAF inhibitors [11–13, 15], we first examined the mutation status and expression of this tumor suppressor (Figure 1D, Table S1). Interestingly, all four non-responder cell lines had aberrant PTEN, which resulted in activation of AKT signaling. Conversely, we did not observe a clear correlation between PTEN wild type status and sensitivity to SCH984. Two of the responder cell lines had genetic alterations in PTEN (WM1552C and WM278) with modest AKT activation. Additionally, two of the four intermediate cell lines (1205Lu and WM793) had hemizygous PTEN deletion and did not express PTEN, whereas the other two intermediate cell lines (WM46 and WM1232) harbored a hemizygous PTEN deletion but still expressed PTEN protein. These observations suggest that even though PTEN may contribute to poor response to SCH984, it may not be sufficient to confer resistance to ERK inhibition. Indeed, shRNA mediated silencing of PTEN in sensitive cells did not affect sensitivity to SCH984 (Supplementary Figure S2A-F). Consistent with this, murine cells derived from a genetically engineered mouse model of melanoma driven by mutant BRAF-V600E and PTEN deletion [17] were sensitive to SCH984 (IC50 0.15 μM; Amax: −73.6%) (Figure S3A-B).

We next selected two responder cell lines, with particularly high Amax and low IC50 and two non-responder cell lines with low Amax and high IC50 to assess the effects of SCH984 on key effectors of the BRAF/MEK/ERK pathway in a time and concentration dependent manner (Figure 1E). SCH984 inhibited the phosphorylation of the ERK effector p90 ribosomal S6 kinase (pRSK) in both responder and non-responder cells; although pRSK baseline levels were lower in the non-responder group. Further, SCH984 inhibited the phosphorylation of the ribosomal protein S6 in the two responder cell lines and one of the non-responder cell lines (WM9), whereas S6 remained phosphorylated in the other non-responder cell line (WM1799). ERK inhibition led to up-regulation of the CDK inhibitor p27KIP1 in both subgroups. Additionally, AKT was phosphorylated in the non-responder subgroup, whereas pAKT levels were relatively low in the responder cells. The pro-apoptotic factor BIM was induced in both subgroups, albeit at lower levels in the non-responder cells. Finally, we detected PARP cleavage and increased apoptosis in responder cells but not in the non-responder cell lines after 72h of treatment with 1 μM of SCH984.

### PI3K and NOTCH signaling are enhanced in non-responder cells

**Figure 2:**
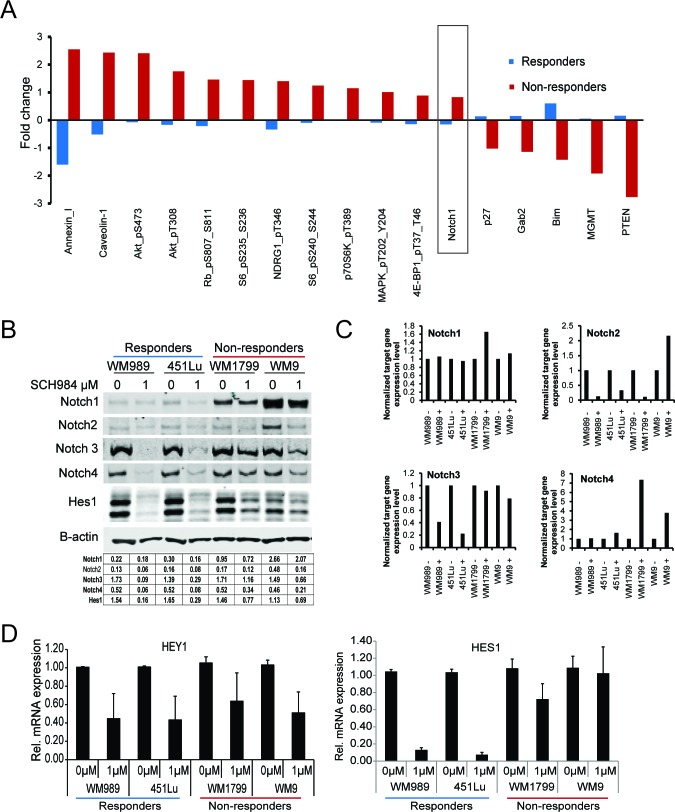
PI3K and Notch signaling are enhanced in SCH984 non-responders **A.** Two responder (451Lu, WM989) and 2 non-responder (WM9, WM1799) melanoma cell lines were treated with 1μM SCH984 for 72h. Total protein lysates were analyzed by reverse phase protein array (RPPA). Proteins with the highest deviation from the global median are shown; mean of three biological replicates. **B.** Two responder and 2 non-responder cell lines were treated with DMSO control or SCH984 1uM for 72h. Immunoblot analysis for Notch1, 2, 3, 4, and Hes1; B-actin was used as loading control. Band intensity was quantified relative to area and B-actin; quantification is shown in table. **C.** mRNA expression levels of *Notch1, 2, 3, and 4* normalized to GAPDH. Relative change between untreated and treated with SCH984 1uM. **D.** *HES1* and *HEY1* mRNA levels were assessed by QRT-PCR in 2 responder and 2 non-responder cell lines.

To determine which regulatory pathways were differentially activated in responder *vs*. non-responder cells, we performed reverse phase protein arrays (RPPA) to analyze the differential expression of 167 proteins involved in signal transduction and melanomagenesis [16]. We assessed the relative expression of total and phospho-proteins in two responder (451Lu and WM989) and two non-responder (WM1799 and WM9) cells after treatment with 1 μM SCH984 for 72h (Figure 2A and Suppl. Figure S4). Most of the top differentially expressed proteins were effectors of the PI3K pathway (Suppl. Figure S5); however, we also noted higher levels of the transmembrane receptor Notch1 in the non-responder cells. These results raised the possibility that enhanced PI3K and/or Notch signaling could be attenuating the response to ERK inhibition. We therefore focused on the transmembrane receptor Notch1, which has not been previously implicated in resistance to MAPK inhibition. Notch1 levels were higher and not substantially affected by SCH984 in non-responder cells compared to responder cells. We also noted that the levels of other Notch family members, including Notch 3 and 4 were inhibited by SCH984 in responder cells, but minimally affected in non-responder cells (Figure 2B). Levels of Notch 2 were relatively low in all cells evaluated except in WM9. We also assess the effect of ERK inhibition on Notch transcripts. We found that treatment of melanoma cells with SCH984 led to decreased Notch2 and Notch3 mRNA levels in responder cells, whereas Notch3 mRNA levels were not affected in the non-responder cells. Transcript levels for Notch 1 and 4 were not affected by SCH984 treatment in the responder cell lines, but Notch4 mRNA increased in the non-responder cell lines (Figure 2C). Additionally, the Notch target genes Hes1 and Hey were downregulated by SCH984 preferentially in the responder cells (Figure 2B, 2D and data not shown).

**Figure 3:**
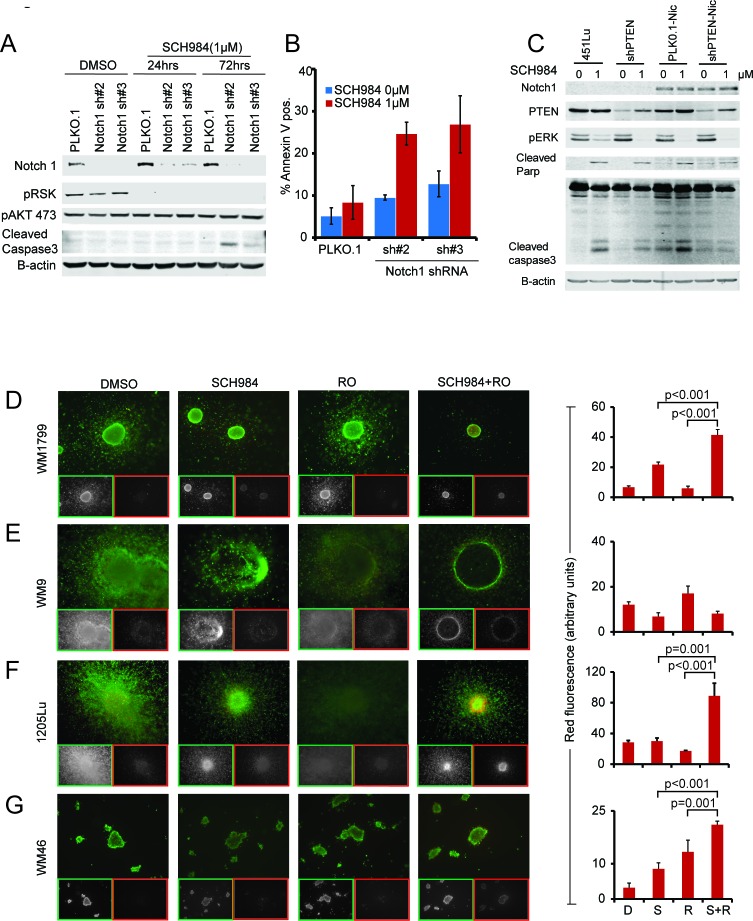
Notch inhibition enhances the effect of SCH984 **A.** The SCH984 non-responder cell line WM9 was transduced with lentiviruses encoding control or two different Notch1 short hairpins; immunoblot of Notch1 confirmed knock down. Cells were treated with SCH984 1uM for 24 and 72h. Protein lysates were probed for pRSK and pAKT as surrogates for MAPK and PI3K pathway activity, cleaved caspase 3 was used to assess apoptosis, B-actin was included to ensure equal loading. **B.** Annexin V staining of control and shNotch1 knock down cells; data show mean of 3 experiments +/− SEM. **C.** The SCH984 responder cell line 451Lu was transfected with vector control, shPTEN, NIC or shPTEN + NIC. Transduced cells were treated with SCH984 1μM for 24 or 48 hours. Protein lysates were analyzed by immunoblotting with the indicated antibodies. Cleaved caspase-3 levels, indicative of apoptosis, were quantified relative to B-actin. **D.**-**G.** Collagen-embedded melanoma spheroids were treated as indicated, stained with Calcein-AM (live cells, green) and EtBr (dead cells, red) and imaged using an inverted microscope. Merged and pseudo-colored images for each condition as well as individual Ca-AM (live; green frames) and EtBr (dead; red frames) are shown. For combination treatment, cells were pre-treated with RO for 24h, followed by RO+ SCH984 for an additional 72h. Cell death was quantified as the average EtBr signal intensity across each spheroid core after removal of background signal; data show average of three independent experiments +/− SEM.

We next asked if silencing Notch1 would sensitize non-responder cells to SCH984. Indeed, shRNA-mediated Notch1 silencing increased apoptosis of cells treated with SCH984 as evidenced by an increase in cleaved caspase-3 and Annexin V staining (Figure 3A and 3B). Concurrent expression of a constitutively activated mutant form of Notch1 (NIC) with PTEN shRNA protected responder cells from SCH984-induced apoptosis (Figure 3C). Likewise, ectopic expression of NIC along with loss of PTEN conferred resistance to the BRAF inhibitor vemurafenib (Suppl. Figure S6). These results indicate that Notch activation could diminish MAPKi-mediated apoptosis in BRAF-V600E mutant cells with aberrant PTEN and raised the possibility that targeting Notch may restore sensitivity to ERK inhibition.

### Gamma secretase inhibitors cooperate with SCH984

**Figure 4:**
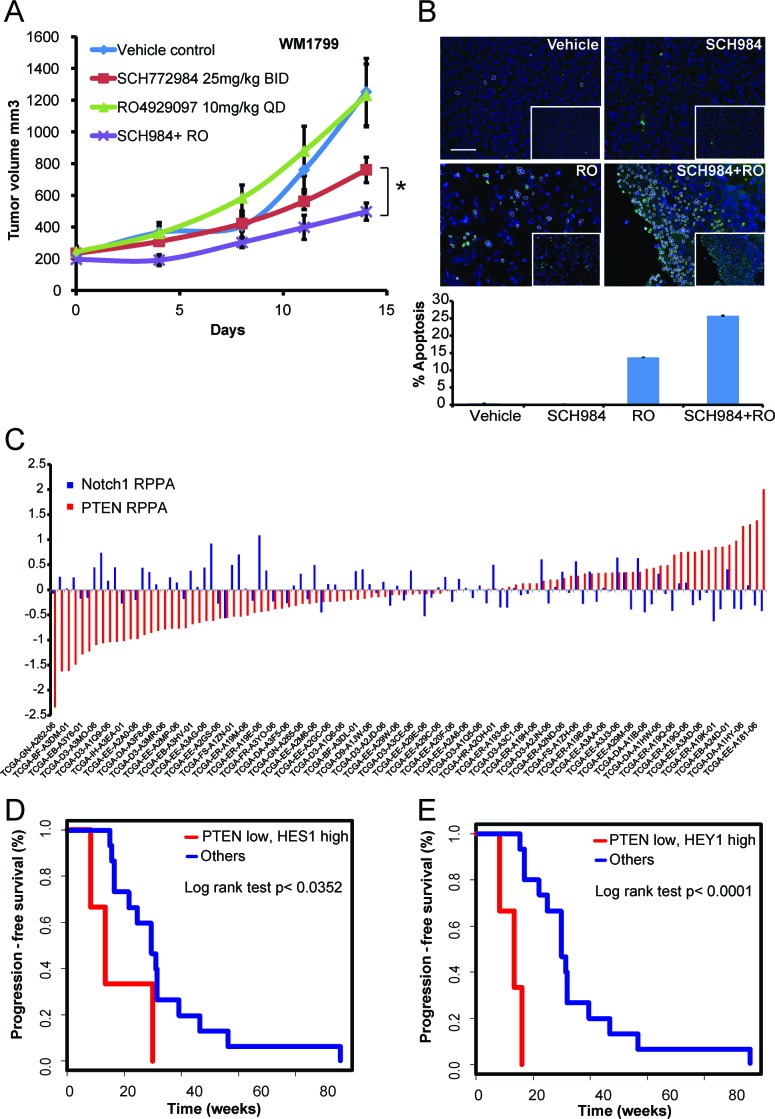
PTEN low and Notch high melanoma patients respond poorer to MAPK inhibitors **A.** The SCH984 non-responder cell line WM1799 was established as xenografts *in vivo*. Mice were randomized into four groups and treated as indicated. The difference in growth rate between SCH984 monotherapy and the combination of SCH984 with RO was statistically significant (*p* = 0.001). **B.** TUNEL staining of representative tumors from vehicle, SCH984, RO, and combination-treated groups harvested 4 hours post last dosing. Images were pseudo-colored to indicate apoptotic cells; original images are shown in the inserts. **C.** RPPA analysis of the TCGA melanoma data for PTEN and Notch1 levels in the BRAF-V600E mutant cohort (*n* = 104). Deviation from the global median is shown. PTEN and Notch1 levels were inversely correlated (Spearman's *r* = −0.2871, *p* = 0.0031). **D.**-**E.** Progression free survival in a set of 21 BRAF-V600E mutant melanoma patients treated with BRAF inhibitors determined by RECIST criteria [43] for patients in clinical trials or by objective response as determined by the treating physician for patients not in clinical trials. Patients with low PTEN/high Hes1 (*p* = 0.0352) or low PTEN/high Hey1 (*p* < 0.0001) have significant shorter progression-free survival than other patients.

To evaluate the value of co-targeting Notch and ERK, non-responder cells grown as 3D-collagen embedded spheroids were treated with SCH984 and the gamma secretase inhibitor RO4929097 (GSI, RO) [17]. Gamma secretase inhibitors block the release of Notch from the membrane thereby inhibiting activation of the Notch pathway [18, 19]. Two non-responder (Figure 3D, 3E) and two intermediate (Figure 3F, 3G) cell lines were selected based on their ability to form spheroids. Whereas treatment with SCH984 or RO as single agents did not substantially affect viability (green stain) of the non-responder cells, SCH984 partially decreased the size and viability of the intermediate 1205Lu spheroids, without substantially inducing cell death (red stain). Conversely, the combination of SCH984 and RO decreased viability and increased apoptosis. Of note, WM9 spheroids were largely hollow and although apoptotic cells were not detected, there were very few viable cells remaining in the combination treatment. Furthermore, in a xenotransplantation model derived from the non-responder cell line WM1799, RO potentiated the effect of SCH984 when the two drugs were used in combination, whereas RO alone had no effect (Figure 4A). SCH984 monotherapy decreased tumor growth 1.6 fold, while the combination of SCH984 and RO at relatively low doses led to a 2.5 fold decrease in tumor volume. The rate of tumor growth was significantly slower in the combination treatment group than in the SCH984 monotherapy group (*p* = 0.001). We used a previously reported dose of RO [18] and did not observe any overt toxicities with either compound alone or in combination. While Notch inhibition induced some apoptosis, the combination of RO plus SCH984 further increased apoptosis in the tumor grafts (Figure 4B). These results indicate that targeting Notch could sensitize melanoma cells to MAPK inhibition. Altogether, our results suggest that concurrent loss of PTEN and enhanced NOTCH activity modulate the response of BRAF-V600E to ERK inhibition.

### PTEN and increased Notch signaling is associated with poor response to MAPK inhibition in BRAF-V600 melanoma patients

Analysis of 104 BRAF mutant tumors in the melanoma TCGA data set showed that PTEN and Notch1 protein levels were inversely correlated (Spearman's *r* = −0.2871, *p* = 0.0031), whereas there was no significant correlation in NRAS mutant melanomas (*n* = 56, Spearman's *r* = −0.0832, *p* = 0.54), or tumors lacking BRAF or NRAS mutations (*n* = 48, Spearman's *r* = −0.1527; *p* = 0.3) (Figure 4C and Suppl. Figure S7).

We then analyzed progression free survival (PFS) on BRAF inhibitor in a set of 21 BRAF-V600E melanoma patients [20] to determine the relationship of aberrant PTEN and Notch activation with clinical response. Samples from BRAF inhibitor treated patients were used as surrogate of MAPK inhibition as ERK inhibitors are in early clinical trials and such data is not available yet. Low PTEN in conjunction with high Notch target gene expression (Hes1 or Hey1) compared to the median was significantly associated with shorter PFS in this cohort (Figure 4D-4E). The median PFS in the Hey1 high/PTEN low group was 12.6 weeks (SEM 2.3 weeks). In contrast the median PFS in the Hes1 high/PTEN low group was 17.2 weeks (SEM 6.5 weeks), far lower than the median PFS of the “other” group at 29.9 weeks (SEM 6.6 weeks, *p* < 0.0001 and *p* = 0.0352 respectively). These data suggest, that aberrant PTEN and Notch activation may attenuate the response to MAPK inhibition in a subset of BRAF-V600E melanoma patients.

## DISCUSSION

Despite recent advances in melanoma treatment, several challenges remain. For example, whereas 70% of BRAF mutant melanomas respond to MAPK pathway inhibitors, a substantial subset is intrinsically resistant. Hence, it is critical to identify molecular determinants of response to inhibitors of the MAPK pathway to optimize the therapeutic benefits of these pharmacological agents. This knowledge will be valuable in selecting the right patient population and devise effective combination therapies that can overcome resistance and enable durable responses.

ERK has emerged as a promising target for melanoma, particularly for melanomas refractory to BRAF and MEK inhibitors using novel ERK inhibitors such as SCH772984 [8], or its clinical analog MK-8353. BVD-523 and GDC-0994, elicit cytotoxic effects in BRAF-V600E melanomas [10]. However, not all BRAF-V600E melanomas respond to these drugs equally; we found that the PI3K and Notch pathways were differentially activated in responder *vs.* non-responder melanomas. While the activation of the PI3K pathway and loss of PTEN have been previously linked to resistance to BRAF or MEK inhibitors [11–13, 15], we found that neither were sufficient to render BRAF-V600E melanoma cells resistant to SCH984. Consistent with our findings, Xing et al. reported that concurrent inactivation of the tumor suppressors PTEN and Rb were required to diminish the dependency of melanoma cells on MAPK activity [12]. However, our non-responder cells expressed functional Rb, suggesting that Rb loss is not required to confer resistance to ERK inhibition. Likewise, Held and colleagues [21] reported that ~25% of BRAF-V600E melanomas are intrinsically resistant to vemurafenib in the context of normal PTEN and Rb expression, supporting the notion that loss of these tumor suppressors is not indispensable for intrinsic drug resistance. Finally, in a recent report examining samples from patients enrolled in the phase I study of the BRAF inhibitor dabrafenib, it was found that copy number changes in *CDKN2A*, *CCND1*, and *PTEN* correlated with PFS [15]. However, this correlation was not statistically significant. Altogether, these findings suggest that other factors besides PTEN loss could be diminishing the response to ERK inhibitors.

We noted that Notch1 levels were high and minimally affected by ERK inhibition in non-responder cells; baseline Notch1 levels were lower in responder cells. Responder and non-responder cells both expressed Notch3 and Notch4; SCH984 decreased Notch3 and 4 expression in the responder cells, while it had marginal effects on Notch3 and 4 levels in the non-responder cells. These results raised the possibility that Notch signaling could be attenuating the cytotoxic effects of SCH984 in the context of aberrant PTEN and PI3K activation. Considering these findings and the fact that PI3K inhibitors tend to have dose limiting toxicities, we explored Notch inhibition as an alternative combination strategy to sensitize melanomas to SCH984. We found that blocking Notch signaling with a gamma secretase inhibitor, potentiated SCH984-mediated tumor cell killing in 3D spheroid assays and in a xenograft model of melanoma. Importantly, the Notch signaling pathway is frequently deregulated in many cancers, including melanoma [22–24]. Notch signaling increases tumor cell proliferation and promotes tumor survival leading to poor patients' outcomes in solid tumors [25–29]. At the molecular level, ligand binding activates Notch signaling by releasing the Notch intracellular domain, which then translocates into the nucleus and activates gene transcription. Wajapeyee and colleagues further linked Notch with melanoma development and progression through a downstream effector of MAPK, miR-146a [30]. Additionally, recent studies have demonstrated that Notch signaling promotes chemo-resistance [31]. Therefore, these and other studies [18, 19] imply that blocking the Notch pathway, likely in combination regimens, could offer a promising therapeutic approach for melanoma.

It has been shown that PI3K/AKT upregulates Notch1 in an NFkβ-dependent manner [22]. Overexpression of Notch can also activate the PI3K/AKT and MAPK pathways [23]; hence, these pathways likely interact via a feedback regulatory loop yet to be fully characterized in melanoma. Although the mechanisms by which Notch decreases dependency on the MAPK pathway need to be explored, some possibilities may be directly related to the ability of Notch to upregulate a number of cytokines, growth factors, and growth factor receptors, including IL-7, NGR1, and IGF-1R, which have been implicated in resistance to BRAF and MEK inhibitors [13, 32–34]. Notch, cytokines, and RTKs can stimulate MAPK, PI3K, mTOR, and NFkβ signaling [35], thereby attenuating the response to SCH984. Crosstalk among these pathways has been reported in preclinical models and may very well operate in a number of human tumors [36]. Additionally, it has been proposed that Notch can induce RAS signaling [37], thus promoting tumorigenesis and potentially resistance to ERK inhibition. Moreover, Notch can activate a transcription program that can conceivably lead to drug resistance [38]. Considering the critical role of this receptor in regulating proliferation and survival in melanoma, further studies evaluating combination therapies targeting Notch are warranted. In our studies, the combination of SCH984 and GSI cooperated to inhibit tumor growth; however, these two compounds at the doses used did not cause tumor regression. This could be in part related to the potency of the available GSI. This class of compounds is associated with GI toxicities, making effective dosing regimens challenging. Hence, it is possible that with the advent of better drugs targeting Notch, anti-tumor effects could be improved [31]. Also, considering the narrow therapeutic index of both ERK and GSI, studies evaluating intermittent or sequential dosing schedules would be needed to diminish potential toxicities. Further, as Notch signaling is context dependent [39], the use of GSI, like other kinase inhibitors, could have some shortcomings, which will need to be closely monitored. The utility of Notch as a potential marker of response to MAPK inhibition deserves broader evaluation in melanoma. Future studies validating the role of Notch as a marker of clinical response could help selecting patients who should be considered for first line combinatorial approaches targeting these pathways.

## MATERIALS AND METHODS

### Cell lines, viability assays, and small molecule inhibitors

Human patient-derived melanoma cell lines were cultured in DMEM medium supplemented with 5% fetal bovine serum and grown at 37°C in 5% CO2. All cell lines were periodically authenticated by DNA finger printing using Life Technologies AmpFISTR Identifier microsatellite kit and tested for mycoplasma by Lonza Mycoalert Assay. Genomic DNA was analyzed for mutations in BRAF, PTEN, NRAS, KIT, CDKN2A, RB, TP53 by the nucleotide extension assay using the iPlex platform (Sequenom, Inc, San Diego, CA) as previously described [40]. RO4929097, PLX4032, PD0325901, AZD8055, GDC0941, BEZ235 were obtained from SelleckChem (Houston, TX). Cell Viability was determined using MTS assays as previously described [32]. IC50 values and maximum inhibitory activity (Amax) were calculated using GraphPad Prism 5 (GraphPad Software Inc.). Amax is defined as the maximal activity of the compound and measures its efficacy in reducing viability.

### NOTCH-1 knockdown using lentiviral-mediated NOTCH-1 shRNA

Knock down of Notch 1 in WM1799 and WM9 was performed using lentiviral particles expressing Notch1-shRNA or control PLKO.1 (Open Biosystems). Lentivirus were produced in 293T cells transfected with lipofectamine 2000 (Invitrogen) in OPTIMEM medium (Gibco, Life Technology) as previously described [41]. Viral supernatants were harvested at 48 and 72 h post transfection. Target cells were transduced with lentiviruses carrying Notch1 shRNA (TRCN0000003359 & TRCN0000003360)) or non-targeting shRNA in the presence of 8 μg/ml polybrene (Sigma) for 24 hrs. Transduced cells were selected with 0.2 μg/ml Puromycin (Gemini Bio-products; cat# 400-128P). Three days post transduction cells were lysed using NP-40 lysis buffer with 100mM Na Vanadate and 1X protease inhibitors (Roche Diagnostics). 30ug of total protein lysate for PLKO.1 shRNA infected and NOTCH 1 shRNA infected cells were loaded on 10% SDS-PAGE. Notch1 expression levels were determined by immunoblotting using Notch 1 antibodies (Cell Signaling; cat # 4380S; 1: 1000 dilution).

### Reverse phase protein analysis (RPPA)

RPPA was performed on cells treated with 1 μM SCH984 for 72h. Cells were lysed with 200ul ice-cold lysis buffer (1% Triton X-100, 50mM HEPES, pH 7.4, 150mM NaCl, 1.5mM MgCl2, 1mM EGTA, 100mM NaF, 10mM Na pyrophosphate, 1mM Na3VO4, 10% glycerol, freshly added protease and phosphatase cocktail tablets (Roche Applied Science Cat. # 05056489001 and # 04906837001). After two flash freeze cycles, samples were centrifuged at 13000rpm for 15 minutes at 4°C and supernatants were collected. Protein concentration was determined by Bro-Rad protein assay (Cat # 500-0006). About 40μl cell lysate (protein adjusted to 1-1.5μg/μl) were mixed with 4X SDS sample buffer (40% Glycerol, 8% SDS, 0.25M Tris-HCL, pH 6.8; beta-mercaptoethanol at 1/10 of volume without bromophenol added before use). The samples were then heated for 10 minutes at 100°C in a heat block and submitted for RPPA processing. RPPA was performed by the MDAnderson Center RPPA core facility as previously described [16] and data reported as Normalized Log2.

### Immunoblotting

Immunoblot analyses were performed as previously described (29). Xenograft tumors were snap frozen in liquid nitrogen immediately after harvesting. Tumor chunks were ground on liquid N2 using a MM2 mixer mill (Retsch, Newtown, PA). Cells and tissue were lysed and equal amounts of protein (10-40μg) were subjected to SDS-PAGE and proteins transferred onto PVDF membranes (Immobilon). Membranes were probed with primary antibodies (Cell Signaling: Erk (#9102, 1:2000), pErk (#4370, 1:1000), pAkt (S473) (#4060, 1:1000) pAkt (T308)(#4056, 1:1000) AKT (#4685, 1:1000) Pten (#9188, 1:1000) pRSK (#9344, 1:1000) Rsk (#8408, 1:1000) pS6 (#2215, 1:1000) S6 (#2217, 1:1000) P27 (#3686, 1:1000) Bim (#2819, 1:1000) Parp (#9542, 1:1000) Notch1 (#4380, 1:1000) Caspase 3 (#9662, 1:1000) cleaved Caspase 3 (#9664, 1:1000) 4EBP1 (#9644, 1:2000) pP70S6K_T389 (#9234, 1:1000) Rb (#9309, 1:1000) Sigma: Actin (#A5441, 1:5000) Vinculin (#SAB4200080, 1:2000) Abcam: Notch2 (#ab8927, 1:1000) Notch3 (#ab23426, 1:1000) Santa Cruz Biotechnology: Notch4 (#SC-5594, 1:1000) Millipore: Hes1 (#AB5702, 1:1000).) overnight at 4°C in TBS-T, then incubated with Alexa Fluor-labeled secondary antibodies (IRDye 680LT goat-anti mouse, IRDye 800CW goat-anti rabbit, donkey-anti mouse, donkey-anti rabbit, or donkey-anti goat IRDye 800CW or 680LT (LI-COR, Lincoln, NE) for 1h and scanned using the Odyssey system (LI-COR, Lincoln, NE).

### Flow cytometry

Melanoma cells were fixed in 70% ethanol and stained with propidium iodide as previously described [32]. For apoptosis quantification, samples were stained with an annexin V allophycocyanin conjugate (Invitrogen, Carlsbad, CA) as previously detailed in [32]. Samples were subsequently analyzed with an EPICS XL (Beckman-Coulter) apparatus.

### Collagen-embedded melanoma spheroids

Melanoma spheroids were generated as previously described [42]. Briefly, 5000 cells/well in 96-well plates were allowed to coalesce for 72h on a non-adherent agar layer before incorporation in a collagen type I matrix. Spheroids were stained with the Live/Dead cell assay (Invitrogen) then imaged using a Nikon Inverted TE2000 microscope (Melville, NY). Images were analyzed using the ImagePro software (Media Cybernetics, Rockville, MD). Unmodified greyscale images were analyzed using the same parameters across all samples. Signal values were obtained using the formula: (Fluorescence value-area count)/Background.

### *In vivo* studies

All animal experiments were performed in accordance with institutional guidelines. NSG mice were inoculated s.c. with 1×10^6^ human melanoma cells in a 1:1 suspension of matrigel (BD Matrigel™) / complete media. When tumors reached a mean volume of 200mm^3^, mice were randomized into 4 treatment groups (10 mice/group). Groups were treated twice daily with vehicle control, SCH772984 25 mg/kg, RO4929097 10mg/kg or the combination of both drugs for 14 days. Tumor size was assessed twice weekly by caliper measurement (length x width^2^ / 2). After 14 days, mice were sacrificed and tumors were harvested four hours after the last dose. Tumor samples were snap frozen in liquid nitrogen for subsequent analysis.

TUNEL (TdT-mediated dUTPnick end labeling) immunohistochemistry was performed on FFPE samples according to the manufacturer's protocol (Roche Diagnostics). Briefly, sections were incubated with the TUNEL reaction mixture for 1h in a humidified chamber at 37° degrees, counterstained with DAPI nuclear stain and imaged by fluorescence microscopy. Only comparable sections of the outer layers of the tumor without signs of necrosis or physically damaged cell morphology in H&E staining were analyzed.

### Statistical analyses

Statistical significance was determined using two-sided Student's *t*-test. *P* < 0.05 was considered to be significant. For *in vivo* experiments, the trends of mean tumor volume over time were compared between xenograft treatment groups using linear mixed models. A likelihood ratio testing nested model was used to examine if trends were overall significantly different among groups. Log-rank test was used to analyze survival data. *P* < 0.05 was considered as significant difference in survival between two groups.

## SUPPLEMENTARY MATERIALS FIGURES AND TABLE


