# Supplementary material for: Targeting Notch enhances the efficacy of ERK inhibitors in BRAF-V600E melanoma
Source: Oncotarget. 2016 Sep 16;7(44):71211–22. doi: 10.18632/oncotarget.12078 (PMC5342073; doi:10.18632/oncotarget.12078)
Supplement: Supplementary file 1 [file oncotarget-07-71211-s001.pdf]

Targeting Notch enhances the efficacy of ERK inhibitors in BRAF-V600E melanoma

Supplementary Material

Supplementary Figure S1

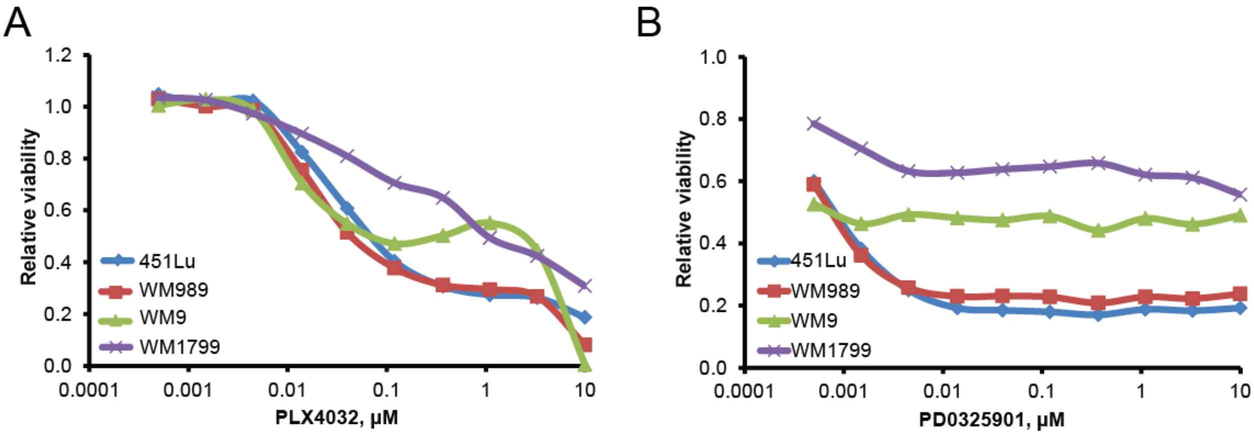

Supplementary Figure S1: Differential response to BRAF and MEK inhibition. A-B. Two responder (451Lu, WM989) and two non-responder (WM9, WM1799) cell lines were treated with the indicated doses of PLX4032 (A) or PD0325901 (B) for 72h. Viability was assessed by MTT assays.

Supplementary Figure S2

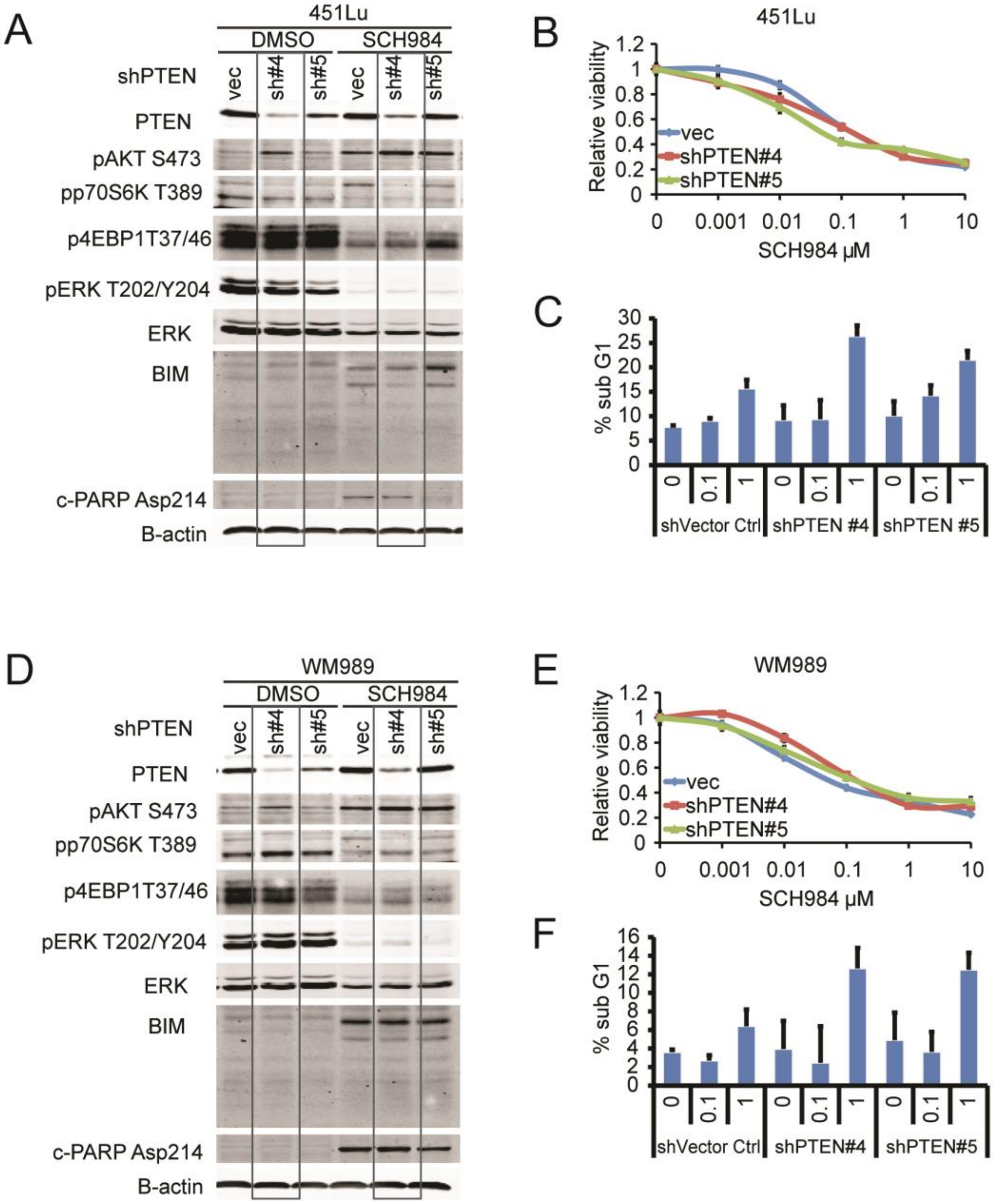

Supplementary Figure S2: Effect of PTEN knock down on sensitivity to ERK inhibition. A. 451Lu cells expressing PTEN shRNA (sh#4, sh#5) or vector control were treated with SCH984 1µM for 48h. Protein lysates were analyzed by western blot. **B.** Vector (vec) and PTEN shRNA- transduced cells were treated with increasing doses of SCH984 for 48 hours. Viability was assessed by MTT assay; one representative experiment (n=7 replicates) +/- SD is shown. **C.** 451Lu cells were stained with propidium iodide (PI) and examined by flow cytometry. Percent of cells in sub G1,

indicative of apoptosis, is shown. Data shown depict mean of three experiments. **D.** WM989 cells were transduced with PTEN shRNA and analyzed as in A. **E.** Vector (vec) and PTEN shRNA- infected cells were treated with increasing doses of SCH984 for 48 hours. **F**. WM989 451Lu cells were stained with propidium iodide (PI) and examined by flow cytometry.

Supplementary Figure S3

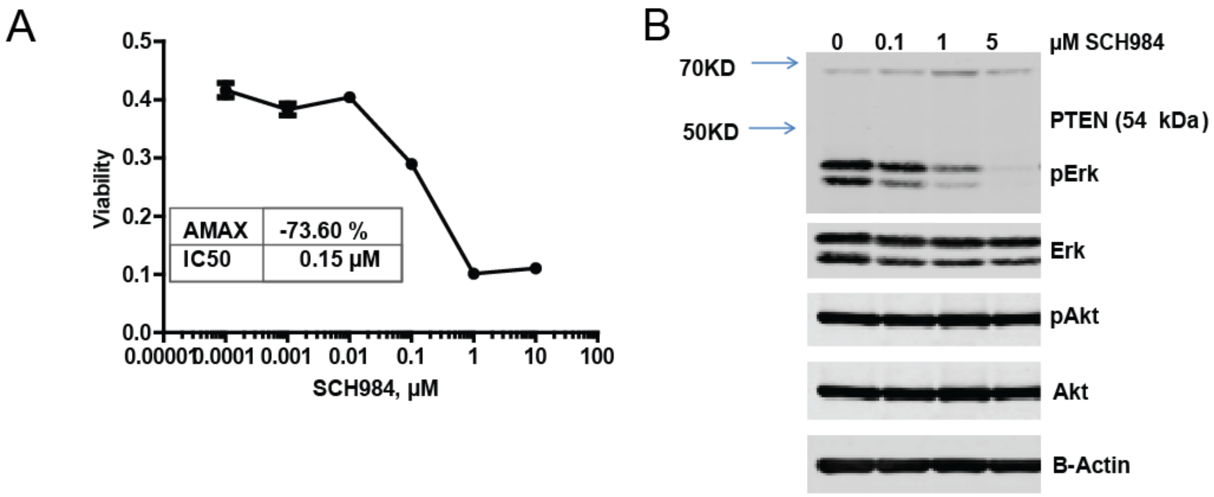

Figure S3: Effect of ERK inhibition in a BRAF V600E and PTEN DEL murine melanoma cell line. **A.** BPC1 (BRAFCA/PTEN-/-) mouse melanoma cells were treated with increasing doses of SCH984 for 72h and viability was assessed by MTS assays.

B. Cells were treated with the indicated doses of SCH984 for 24h. Protein lysates were analyzed by western blot.

Supplementary Figure S4

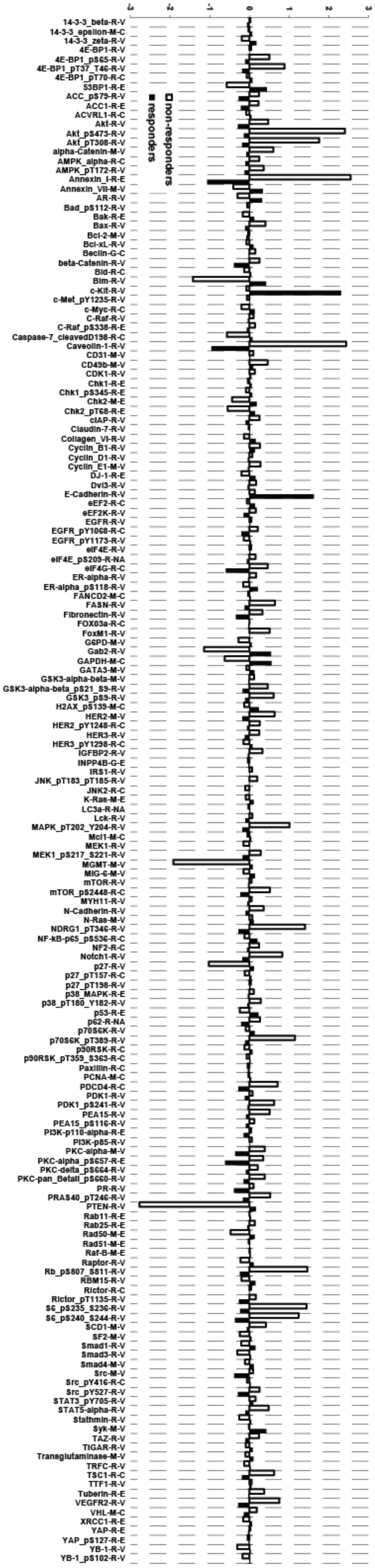

Supplementary Figure S4. Analysis of complete RPPA data set. RPPA analysis of lysates from four melanoma cell lines treated with 1µM SCH984 for 72h. Deviation from the global median is shown for all proteins probed. Data shown are mean of three replicates from 2 responder (451Lu, WM989) and 2 non-responder cell lines (WM9, WM1799).

Data can be accessed at:

https://dl.dropboxusercontent.com/u/67811895/Suppl%20Fig%20S4R1.xlsx

Supplementary Figure S5

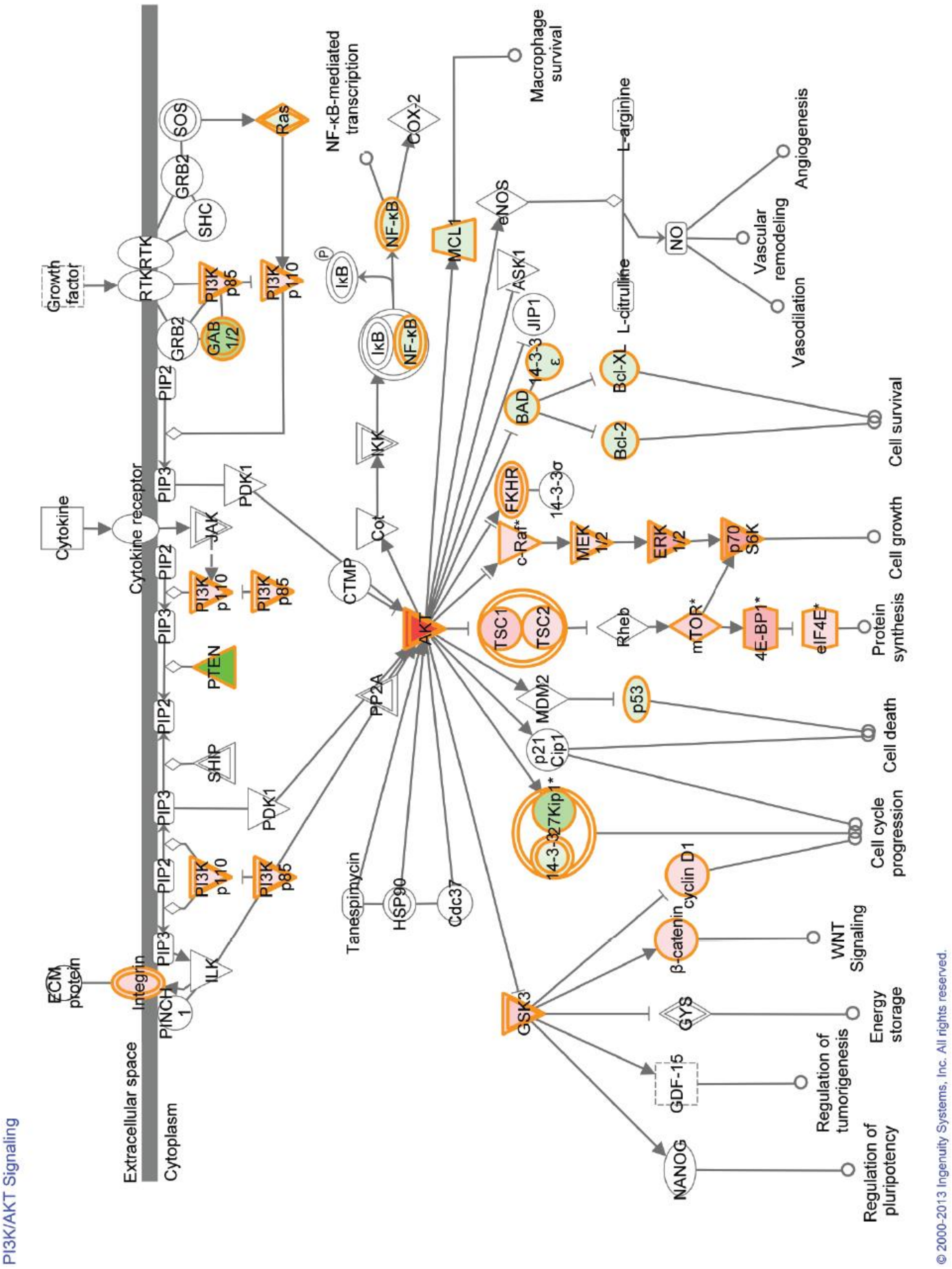

© 2000-2013 Ingenuity Systems, Inc. All rights reserved.

Supplementary Figure S5. Ingenuity pathway analysis of RPPA data for non-responder cell lines. Green indicates proteins that were down-regulated, and red indicates proteins that were upregulated compared to the global mean.

Supplementary Figure S6

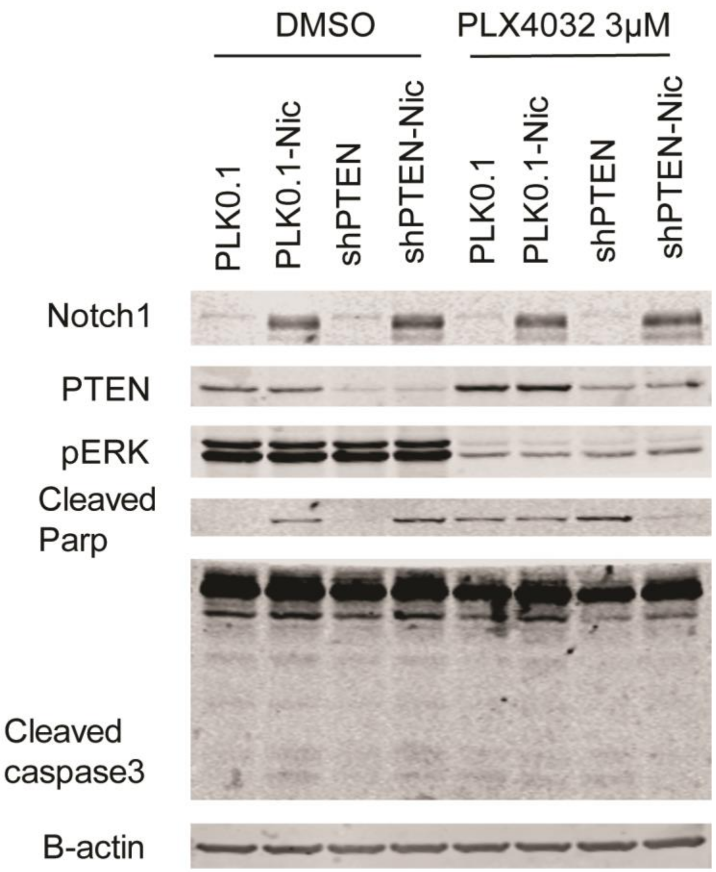

Supplementary Figure S6. Ectopic expression of NIC along with loss of PTEN conferred resistance to the BRAF inhibitor vemurafenib.

Concurrent expression of a constitutively activated mutant form of Notch1 (NIC) with PTEN shRNA protected responder cells from vemurafenib-induced apoptosis

Supplementary Figure S7

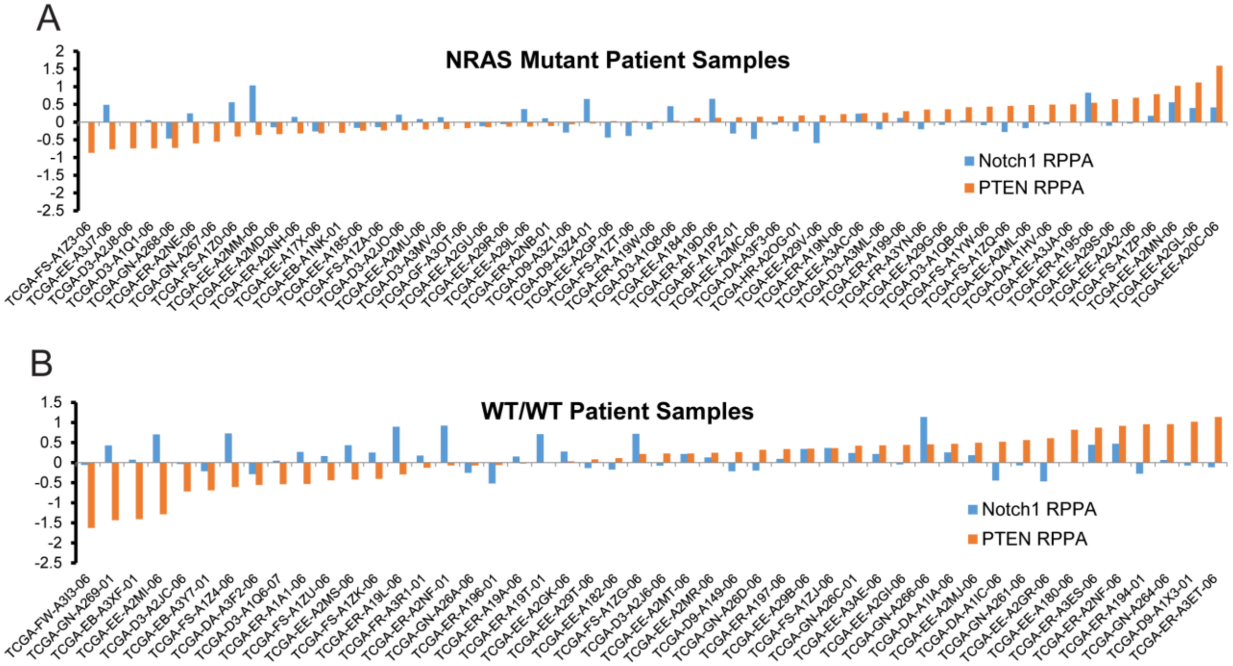

Supplementary Figure S7. RPPA analysis of TCGA melanoma patient samples for PTEN and Notch1. Deviation from the global median is shown for PTEN and Notch1 proteins. **A.** NRAS mutant (n=56, Spearman's r = -0.0832, p=0.54); and **B.** BRAF wild type and NRAS wild type cohort (n= 48, Spearman's r = -0.1527; p= 0.3).

Supplementary Table S1: Cell lines used in the study

Cell line ID	Stage	BRAF	PTEN (MUT/CN)	NRAS	C-KIT	AKT	CDKN2A (MUT/CN)	Rb (MUT/CN)	TP53
WM88	MET	V600E	WT/ND	WT	WT	WT	Homo Del	WT/ND	WT
WM989	MET	V600E	WT/ND	WT	WT	WT	MUT/ND	WT/ND	Y220C
451Lu	MET	V600E	WT/ND	WT	WT	WT	MUT/Hem Del	WT/ND	Y220C
WM983B	MET	V600E	WT/ND	WT	WT	WT	MUT/Hem Del	WT/HemiDel	P278F
WM164	MET	V600E	WT/ND	WT	WT	WT	MUT/Hem Del	WT/ND	Y220C
WM1552C	RGP	V600E	MUT/Hem Del	WT	WT	WT	WT/ND	WT/ND	R248Q
WM278	VGP	V600E	WT/Hem Del	WT	WT	WT	WT/ND	WT/ND	WT
WM46	MET	V600E	WT/Hem Del	WT	WT	3E17K	Homo Del	WT/ND	WT
1205Lu	MET	V600E	MUT/ Hem Del	WT	WT	WT	Homo Del	WT/A	WT
WM1232	MET	V600E	WT/Hem Del	WT	WT	WT	MUT/ND	WT/ND	Y220C
WM793	VGP	V600E	MUT/ Hem Del	WT	WT	WT	WT/ND	WT/ND	WT
WM1158	MET	V600E	WT/Hem Del	WT	WT	WT	MUT/ND	WT/ND	WT
WM9	MET	V600E	WT/Hem Del	WT	WT	WT	WT/Hem Del	WT/ND	WT
WM1799	MET	V600E	WT/Hem Del	WT	WT	WT	Homo Del	WT/ND	WT
WM3704	MET	V600E	MUT/ND	WT	WT	WT	WT/ND	WT/ND	P278F

MET denotes cell lines derived from metastatic melanoma lesions

RGP radial growth phase primary melanoma

VGP vertical growth phase primary melanoma

BRAF, NRAS, C-KIT, and AKT status was assessed using sequenom
PTEN and CDKN2A copy number was assessed by MLPA
PTEN and CDKN2A mutation was assessed by sequencing
RB copy number was assesssed by aCGH
HemDel; hemizigous deletion
A, Amplified
ND, Not determined
MUT, mutant
CN, copy number
